# Italian Patients Journey for Gene Therapy in Haemophilia A

**DOI:** 10.1111/hae.70332

**Published:** 2026-06-15

**Authors:** Giovanni Di Minno, Gaia Spadarella, Alessandra Borchiellini, Anna Borrelli, Andrea Buzzi, Giancarlo Castaman, Michele Cecchi, Raimondo De Cristofaro, Vincenzo La Mura, Angelo Lupi, Giuseppe Malcangi, Maria Elisa Mancuso, Maria Francesca Mansueto, Cristina Santoro, Rita Carlotta Santoro, Luigi Piero Solimeno, Annarita Tagliaferri, Ezio Zanon, Isabella Garagiola, Flora Peyvandi

**Affiliations:** ^1^ Dipartimento Di Medicina Clinica e Chirurgia Federico II University, School of Medicine Napoli Italy; ^2^ Dipartimento Di Scienze Biomediche Avanzate Federico II University, School of Medicine Napoli Italy; ^3^ Regional Centre For Hemorrhagic and Thrombotic Diseases Hematology Unit AOU Città della Salute e della Scienza Turin Italy; ^4^ Direzione Sanitaria AOU “Federico II” Napoli Italy; ^5^ Fondazione Paracelso Milano Italy; ^6^ Center For Bleeding Disorders and Coagulation Careggi University Hospital Florence Italy; ^7^ UOC Farmacia ospedaliera Dell' Azienda Ospedaliero‐Universitaria Careggi Florence Italy; ^8^ Dipartimento Di Medicina e Chirurgia Traslazionale Università Cattolica S. Cuore Rome Italy; ^9^ Servizio Malattie Emorragiche e Trombotiche Fondazione Policlinico Universitario Agostino Gemelli IRCCS Rome Italy; ^10^ Fondazione IRCCS Ca' Granda Ospedale Maggiore Policlinico Angelo Bianchi Bonomi Hemophilia and Thrombosis Center and Fondazione Luigi Villa Milan Italy; ^11^ Department of Pathophysiology and Transplantation Università Degli Studi di Milano Milan Italy; ^12^ Federation of Haemophilia Associations (FedEmo) Milan Italy; ^13^ UOSD Centro Emofilia e Trombosi Azienda Ospedaliero Universitaria Policlinico di Bari Bari Italy; ^14^ Center For Thrombosis and Hemorrhagic Diseases IRCCS Humanitas Research Hospital Milan Italy; ^15^ Humanitas University, Pieve Emanuele Milan Italy; ^16^ Dipartimento Di Oncologia, U.O. Ematologia Policlinico Paolo Giaccone Palermo Italy; ^17^ Dipartimento Di Medicina Traslazionale e Di Precisione, Ematologia Sapienza Università di Roma Rome Italy; ^18^ Hemostasis and Thrombosis Unit Azienda Ospedaliero Universitaria Dulbecco Catanzaro Italy; ^19^ Division of Orthopaedic Surgery and Traumatology Foundation IRCCS Ca’ Granda, Ospedale Maggiore Policlinico Milan Italy; ^20^ Regional Reference Centre For Inherited Bleeding Disorders Parma University Hospital Parma Italy; ^21^ Centro Regionale HUB Multidisciplinare Per La prevenzione, Profilassi e Trattamento Avanzato Dell'artropatia emofilica. Clinica Medica 1° Ad Indirizzo Trombotico‐Emorragico Azienda Ospedale Università Di Padova Padua Italy

**Keywords:** decision making, gene therapy, haemophilia A, models, organizational, patient pathway, shared

## Abstract

**Introduction:**

Gene therapy (GT) provides sustained FVIII levels without repeated infusions in Hemophilia A (HA) patients thus overcoming a major limitation of replacement therapy. However, issues remain e.g., patient selection criteria, duration and variability of transgene expression, quality of life and long‐term safety.

**Aim:**

This expert opinion guidance aims to define the optimal operational pathway and organizational model in Italy for the approved gene therapy (GT) product valoctocogene roxaparvovec, for patients with HA.

**Methods:**

A multidisciplinary Working Group (healthcare professionals experienced in managing patients with HA and representatives of patients’ Associations) critically analysed the Summary of Product Characteristics (SmPC) of valoctocogene roxaparvovec and some key publications concerning GT in haemophilia. The emerging evidence, alongside with the Working Group member expertise, helped to develop this journey for Italian patients with HA treated with this AAV5‐FVIII based GT.

**Results:**

The present journey is divided into five sections: selection and screening of eligible patients, GT prescription, GT administration and post‐infusion (short‐term [≤1 year] and long‐term [>1 year‐up to 15 years] post‐GT follow‐up). Each phase involves collaboration between centres organized in a Hub and Spoke model, adoption of Shared Decision Making (SDM) approach, multidisciplinary team working and national data collection.

**Conclusion:**

This product‐specific operational pathway proposal provides Italian clinicians with a comprehensive framework for managing patients with HA treated with GT, acknowledging the evolving nature of this innovative therapy and the need for continuous evaluation of the proposed pathway. The present journey may need adaptation for other GT products.

## 1 Introduction

Hemophilia A (HA) is a rare congenital bleeding disorder caused by a deficiency of coagulation factor VIII (FVIII).

In 2016, according to the National Registry of Congenital Coagulopathies, there were 4120 patients with HA in Italy, with a prevalence of 13.9 cases per 100,000 males [1].

HA may cause bleeding events, occurring spontaneously or following trauma/surgery/invasive procedures, potentially leading to life‐threatening situations [2, 3, 4, 5]. The severity of the disease is classified based on residual FVIII activity levels as severe (FVIII <1 IU/dl), moderate (FVIII 1–5 IU/dl) and mild (FVIII 5.01–40 IU/dl) [5]. Patients with severe HA are at a high risk of spontaneous or trauma‐related bleeding, commonly represented by hemarthrosis [5, 6, 7].

The cornerstone of treatment for severe HA is intravenous administration of exogenous FVIII, either on a prophylactic or on‐demand basis [5, 8, 9, 10, 11]. However, replacement therapy with FVIII is associated with limitations, including the need for frequent infusions (2–3 times/week) [9], which leads to burden on patients’ daily lives [12] and the potential development of neutralizing alloantibodies (inhibitors) to FVIII, which reduces effectiveness of exogenous factor [13, 14, 15].

Extended half‐life FVIII concentrates, which allow less frequent intravenous infusions [16] and subcutaneous drugs that mimic the action of FVIII [5, 8, 9, 10, 17] have been developed to reduce treatment burden and improve effectiveness. However, none of the currently available options provide sustained stabilization of FVIII to levels that avoid the persistence of residual bleeding risk [12, 18]. Recently, the Ultra‐Extended Half‐Life (UHL) FVIII efanesoctocog alfa has been approved in Europe (but is not yet reimbursed in Italy) for treatment and prophylaxis of bleeding in patients with HA [19]. Although it may approach haemostatic normality, it still requires at least weekly intravenous infusion [20].

By allowing the endogenous FVIII synthesis at levels sufficient to provide effective prophylaxis [13, 21] Valoctocogene roxaparvovec is the first gene therapy (GT) approved for the treatment of HA that provides long‐term protection against bleeding events to HA patients with a single administration [22], that is, without the need for exogenous FVIII replacement therapy. In addition, GT also overcomes the limitations of other HA approaches, consequently improving patients’ quality of life [12, 18, 23].

Despite its potential, several issues associated with the use of GT for HA remain unclear, including patient selection criteria, duration and variability of transgene expression, impact on quality of life and long‐term safety. In addition, the complexity and potential issues related to prescription, administration and monitoring require specific considerations for centres offering GT to ensure maximum benefit and optimal care [18, 24].

Valoctocogene roxaparvovec, a GT vector based on an adeno‐associated virus serotype 5 (AAV5) containing a modified FVIII gene [25], is the first GT approved for the treatment of HA that provides long‐term protection against bleeding events to patients with a single administration [25, 26, 27].

This paper aims to provide an optimal pathway for patients with HA treated with valoctocogene roxaparvovec in Italy by defining the operational and organizational model, from eligibility steps to treatment and follow‐up phases, identifying healthcare professionals involved and their roles, defining the relationship and interaction between centres and proposing actions to facilitate the pathway.

## 2 Materials and Methods

A multidisciplinary Working Group of Italian healthcare professionals involved in the management of patients with HA ‐that also included representatives of patients’ associations‐ was established to propose an optimal management model for these patients when treated with GT. First, the Working Group ‐funded by the product Manufacturer‐ critically analysed the Summary of Product Characteristics (SmPC) of valoctocogene roxaparvovec [28]. Of the numerous gene therapy guidelines already published, the UK gene therapy guidance and the WFH gene therapy recommendations [29, 30] were thoroughly analysed by each member of this panel and discussed together. As clearly stated in the UK guidance, ‘*Proposed regional and national multidisciplinary teams will harmonize clinical practices incorporating expertise from various specialties and professional groups. Key requirements identified include standardized documentation and multidisciplinary collaboration. Nationally agreed patient information and counselling checklists will streamline the informed consent process and facilitate data collection for long‐term safety and efficacy monitoring*’. In addition to the discussion of these two guidelines and their reference list, key published studies in the area [13, 18, 24, 25, 26, 27, 31, 32, 33, 34, 35, 36, 37, 38, 39, 40, 41, 42, 43, 44, 45, 46, 47, 48, 49, 50, 51] identified by the Working Group members based on the individual expertise‐ were thoroughly analysed by each member of the panel and discussed with the other panellists. No major dissent relative to data analysis occurred. The emerging evidence provided the background for the development of the present journey of Italian HA patients treated with AAV5‐gene therapy, an expert opinion guidance focused on the definition of the phases of the process and the healthcare figures involved.

### 2.1 Hub and Spoke Treatment Model

The European Association for Haemophilia and Allied Disorders (EAHAD) and the European Haemophilia Consortium (EHC) have proposed the “Hub‐and‐Spoke” model for the treatment of haemophilia and rare bleeding disorders using gene therapies [42, 52]. According to this model, GT must be prescribed and managed by expert haemophilia comprehensive care centres (Hub) and monitored by haemophilia treatment centres distributed across the territory (Spoke) in close communication with the Hub centre. Table 1 summarizes the criteria for designating Hub‐and‐Spoke centres.

**TABLE 1:** Criteria for designating Hub‐and‐Spoke Centres.

Hub Centre	Staff with experience in the management of congenital bleeding disorders. Multidisciplinary team (including hepatologists, infectious disease specialists, orthopaedic surgeons, hospital pharmacists and psychologists) for routine treatment of complex cases and critical care management, reachable 24 h/7 days. In‐house laboratory, operating or reachable 24 h/7 days. Internal pharmacy adequately equipped for receipt and temperature‐controlled storage of drugs and laboratories for the preparation of sterile galenic formulations according to NBP FU XII ed. Nurses trained for the management of congenital bleeding disorders, emergency care and data managers for data collection. Management of informed consent. Adequate resources and trained personnel for the practical management of gene therapy Clinicians with experience in managing gene therapy. Ward with the capacity for inpatient care.
Spoke centre	Staff with experience in the management of congenital bleeding disorders. Multidisciplinary team (including hepatologists, infectious disease specialists, orthopaedic surgeons and psychologists) for complex cases and critical care management, reachable 24 h/7 days. Coagulation laboratory accredited for pre‐screening and follow‐up indicators measurement (FVIII and liver markers). Staff with the capacity for planning, ordering and managing the measurement of anti‐AAV antibodies (Companion Diagnostic [CDx]).

One of the main criteria is the presence of a multidisciplinary team [5, 12]. The management of haemophilia patients involves many professionals who should actively communicate and collaborate. During GT treatment, it is even more significant to ensure the presence of a multidisciplinary team led by a haemophilia specialist, that includes hepatologists, orthopaedics, psychologists/counsellors, physiatrists/physiotherapists, nurses, hospital pharmacists and laboratory staff. The patient associations play a vital role in supporting individual patients in gene therapy to guarantee continuous communication and collaboration among all involved professionals and Hub and Spoke centres to optimize patient management, accelerate diagnostic and therapeutic choices and ensure timely intervention. The key figures in the GT treatment pathway should be haemophilia specialists. Based on the result of an Italian Delphi consensus, the EAHAD model for the management of HA GT was adopted in Italy [12]. The flexibility of the EAHAD model also allowed stakeholders to identify centralized regional infusion Centres (e.g., Naples, Padua, Milan) and related competencies (e.g., pharmacy, lab) based on (1) former experience in the specific issue and (2) documented skill as coordinating reference Centres.

### 2.2 The Patient Journey

The present pathway of haemophilic patients eligible for GT treatment has been organized into five sub‐processes, represented through a flowchart outlining the stages, actors involved and duration of the pathway:

1. Selection and screening of eligible patients.
2. GT prescription.
3. GT administration and post‐infusion follow‐up.
4. Short‐term post‐GT follow‐up (≤1 Year).
5. Long‐term post‐GT follow‐up (>1 year—up to 15 years).

### 2.3 Selection and Screening of Eligible Patients

The patient selection and screening process is managed by the Spoke Centre (except for patients for whom the Hub Centre serves as the reference centre. In this case, the Hub Centre is responsible for this subprocess) (Figure 1).

**FIGURE 1:**
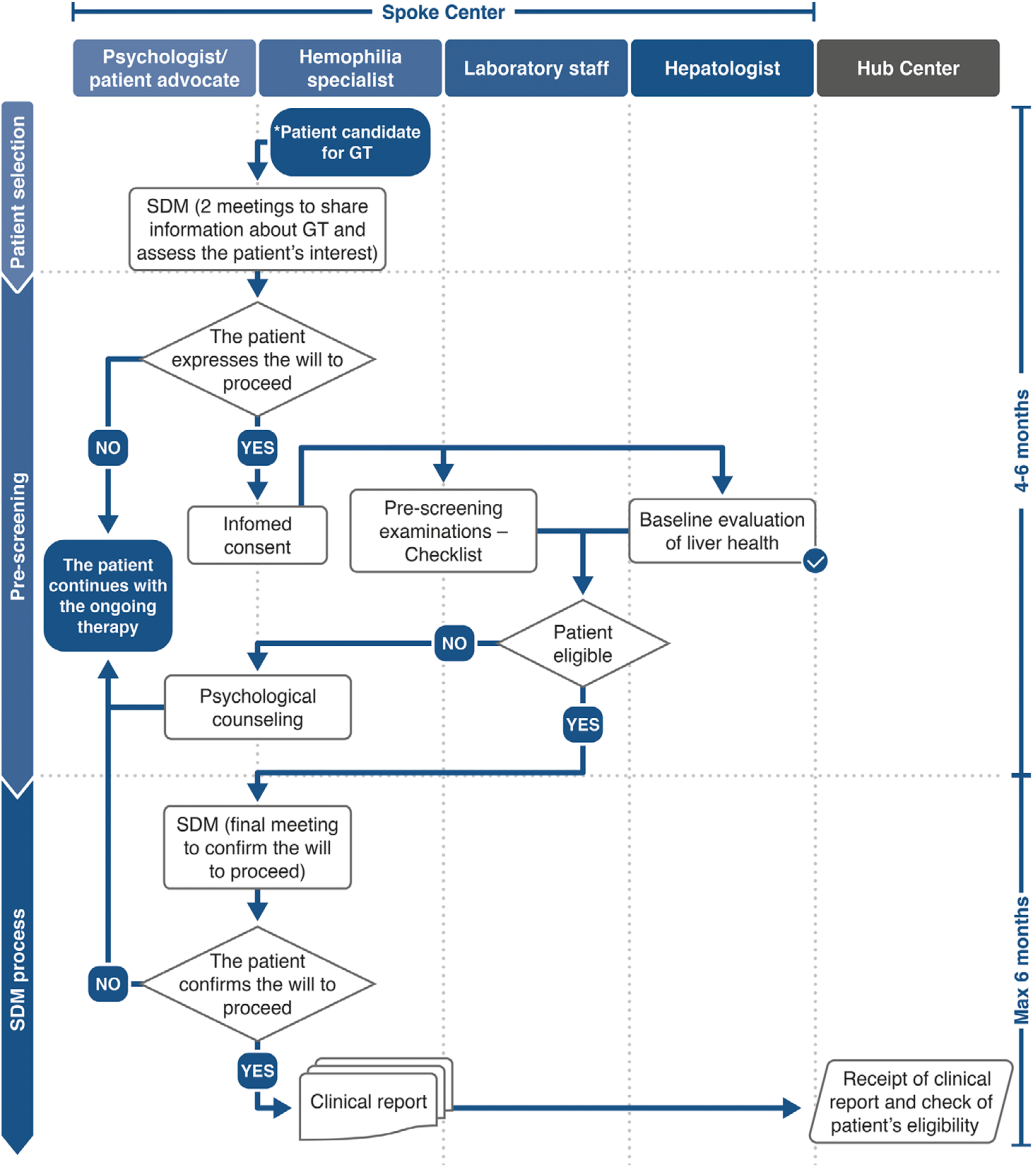
Patient journey: Selection and screening of eligible patients. *Adult patients with severe HA without a history of FVIII inhibitors and without detectable antibodies to AAV5^25^ *Note*: The boxes with the check marks contain indications from the summary of product characteristics. GT, Gene Therapy; SDM, Shared Decision‐making.

During the patient selection phase, the Spoke Centres identify potential candidates for GT: adult patients with severe HA without (1) a history of FVIII inhibitors and (2) detectable anti‐AAV5 antibodies [25]. With respect to the latter issue, anti‐AAV5 antibodies were centrally determined by BioMarin employing the AAV5 DetectCDx, an in vitro diagnostic test to qualitatively reveal anti‐AAV5 antibodies in plasma. AAV5 positivity was defined according to the Manufacturer's recommendations.

*Informed consent*: Especially in a clinical setting like GT the perceived benefits should not minimise the challenges that individuals in whom new genetic material is introduced face. Together with confirming that a haemophilia person has understood the long‐term risks and consequences of GT, meaningful informed consent is critical as to the individual's right to define personal goals and confirm autonomous choices. In Italy, there was no centralized informed consent for GT. However, a model to be employed for local informed consents aimed at protecting patients’ interests, with emphasis on haematological and liver function‐related information has been developed to provide evidence‐based strategies to foster patient‐centred decision support interventions. The need has also been considered to ensure best‐practice recommendations for patient‐physician discussions concerning highly technical and complex therapeutic information.

SDM: A practical guideline based on the awareness of individual goals, preferences and priorities of persons with severe congenital haemophilia undergoing liver‐directed GT, finalized to a tailored shared decision‐making approach has been prepared by the working group [53]. Based on awareness of individual PWH goals, preferences and priorities, a practical guideline has been prepared ‐to be exploited by the clinician‐ to provide updated information to each person with haemophilia during the SDM process; to motivate tailored decisions and maximize psychological support in GT. The key e program identifies five pillars supporting the SDM on gene therapy: (1) pre‐gene therapy information‐seeking; (2) pre‐gene therapy decision‐making; (3) treatment initiation; (4) short‐term post‐gene therapy follow‐up (up to 1 year); (5) long‐term post‐gene therapy follow‐up (>1 year). Based on this guideline‐program, the NHF Medical and Scientific Advisory Council has published a psychosocial assessment template to assess PWH considering GT [54]. In the present experience, during the ‘*pre‐gene therapy information‐seeking*’ stage, clinicians explain what GT is and how it provides a corrected version of a missing or defective gene without directly changing the defective gene within the individual DNA. Special attention is devoted to operative procedures (e.g., administration by a single infusion into a vein, lasting 2–4 h) and how GT works in the body, with comparisons with the usual treatments. Clinicians provide a comprehensive explanation of available GT trials and the effects of GT on the quality of life (e.g., to reduce the regular prophylaxis, or to be able to do more physical activity) should be emphasized [53]. Information on irreversibility of GT, other treatment options, long‐term safety, areas of uncertainty, exact timings of alcohol abstinence and contraception, as recommended in major guidance in the area was also provided [29, 30]. During the ‘*pre‐gene therapy decision‐making*’ stage, [53] clinicians describe the experience of other people with haemophilia who have undergone GT, highlighting that many PWH experienced reduction in bleeds and have stopped regular prophylaxis. Short‐term risks (i.e., immediate reactions to infusion or possible temporary liver changes) and side effects (i.e., weight gain, nausea, high blood pressure, risk of infection, troubles in sleeping) is also provided [53]. In the ‘*Treatment initiation*’ stage ‐together with thoroughly discussing precautions and issues in PWH using emicizumab [55], clinicians educate PWH on how to identify if and when the treatment has worked and monitor the bleeding risk changes after GT; when to stop the regular prophylaxis (≈after 4‐weeks, i.e., when circulating levels of endogenously synthesized FVIII are ≥ 5U/ml) and how to modify specific correlated behaviours (i.e., drink alcohol behaviour, condom use). Concerning the ‘*Short‐term post‐gene therapy follow‐up (up to 1 year)*’ stage, clinicians illustrate the time schedule of controls and emphasize that the most frequent monitoring would be in the first three months, with regular visits through the first year. Emphasis is also devoted to temperature monitoring, pulse, breathing and weight rates, laboratory tests (e.g., blood, urine and semen samples), heart health and joint assessments, to side effects recording, quality of life questionnaires (with emphasis on the HAEMO‐QoL‐A, i.e., the haemophilia‐specific quality of life questionnaire for adults used in this setting) and bleed diary revision. With respect to the *‘Long‐term post‐gene therapy follow‐up (more than 1 year)’* stage, clinicians describe what is known about long‐term safety and requirements for long‐term follow‐up, registry participation and other long‐term needs. Treatment options if factor expression drops too low to reliably offer protection should be provided.

Once the patient gives informed consent, the Spoke Centre verifies the patient's eligibility for treatment through:

- Medical history collection to assess ongoing therapies (e.g., anticoagulants), absence of autoimmune and chronic liver diseases, clinical history of neoplasms or FVIII inhibitors and presence of joint prostheses.
- Assessment of haematological (within one month of infusion) and immunological (anti‐AAV antibody) measurements to identify potential candidates for GT (within three months of infusion and repeated ∼1 month before), liver, kidney and joint health markers (the patient‐specific assessment of joint health within four months of infusions) (Table 2).

**TABLE 2:** Pre‐screening checklist.

	Indicators				
	Haematological	Immunological	Joint health	Liver health^b^	Kidney
**As per SmPC** [28]	‐ FVIII^a^	‐ Anti‐AAV antibodies (CDx) ‐ FVIII Inhibitors ‐ Anti‐HIV Antibodies	—	‐ ALT ‐ AST ‐ GGT ‐ Total bilirubin ‐ Spontaneous INR	—
**Recommended by the working group**	‐ Complete Blood Count including platelets ‐ Inflammation Markers ‐ Electrophoretic Profile ‐ Metabolic markers (cholesterol levels: total, HDL, LDL; Blood Sugar, etc.)		‐ Patient‐specific assessment of joint health through ultrasound examination at least once a year (HEAD‐US) combined with functional evaluations (HJHS). If necessary, refer the patient to an orthopaedic/physiatrist. ‐ Assessment of periprosthetic osteoporosis, with attention to the type of prosthesis, in patients with joint prostheses	‐ aPTT ‐ ALP ‐ Serum Protein Electrophoresis ‐ AFP ‐ Pseudocholinesterase^*^ ‐ Blood glucose, total cholesterol, HDL, triglycerides ‐ Ultrasound and FibroScan ‐ Gamma globulin ‐ Direct bilirubin ‐ HBsAg, HBsAb and HBcAb ‐ HCV‐RNA (in HCV‐Ab positive patients) ‐ HBV‐DNA (in HBsAg‐positive patients) ‐ HDV‐Ab in HBsAg positive patients ‐ HDV‐RNA in HDV‐Ab positive patients	‐ Creatinine Clearance (Estimated) ‐ Urinalysis ‐ Urea

Abbreviations. AFP: Alpha‐Fetoprotein; ALT: Alanine Aminotransferase; aPTT: Activated Partial Thromboplastin Time; AST: Aspartate Aminotransferase; CDx, Companion Diagnostic; GGT: Gamma‐Glutamyl Transferase; HBcAb: Hepatitis B Core Antibody; HbsAb: Hepatitis B Surface Antibody; HBsAg: Hepatitis B Surface Antigen; INR: International Normalized Ratio.

*Pseudocholinesterase, gamma globulin and direct bilirubin help to provide a more comprehensive assessment of liver health and to exclude a possible case of liver disease or shift in liver biochemistry.

^a^ Chromogenic assay.

^b^ Cut‐off values: ALT, AST and GGT according to the reference values of the centre; total bilirubin = 1.25 times the upper limit of normal (ULN); Spontaneous INR ≥ 4.

The assessment should be accomplished by a haemophilia specialist with the support of a hepatologist for liver evaluation and an orthopaedic/physiatrist for joint health evaluation. With respect to the pre‐ and post‐GT psychological assessments, as well as the role of psychologists at different stages of the GT treatment process in this journey, a comprehensive overview ‐that also includes the role of psychologists and the measurement tools used to assess the psychological aspects of individuals undergoing GT‐ has been recently published [56].

The baseline evaluation of liver health is critical to determine the patient's eligibility for GT [53, 57, 58]. In the early stage as well as in the phase 3 study, liver disease was an exclusion criterion [59, 60]. In the SmPC of valoctocogene roxaparvovec [28], GT is contraindicated in patients with uncontrolled acute or chronic liver infections, significant liver fibrosis and known cirrhosis and it is not recommended for patients with other liver conditions and abnormalities in liver laboratory values or a history of liver neoplasms. That a careful pre‐treatment assessment of liver health should include laboratory tests, abdominal ultrasound and liver stiffness measurements by transient elastography (TE) strongly supports the choice of a critical role for hepatologists in the Italian patient journey [61]. Accordingly, health assessments in the Italian patient journey include clinical and laboratory tests to detect the most common risk factors of chronic liver damage (e.g., steatosis, active hepatitis infection). Liver imaging (ultrasound) and liver stiffness measurement (LSM) <8 kPa by vibration‐controlled transient elastography (VCTE; FibroScan; Echosens, Paris, France) is performed to standardize the method and be consistent with the recommended cut‐offs. In case of an uncertain diagnosis, a liver biopsy is required. Moreover, in the first year after therapy, ALT levels are monitored once/week to detect elevations (≥ 1.5 × ULN) that may require immunosuppressant therapy. The issue of liver laboratory values after GT administration ‐with emphasis on Aspartate Aminotransferase (AST) and Alanine Aminotransferase (ALT)‐ is also thoroughly pursued. Current knowledge implies that, depending on the dose and serotype of the vector, the onset of the T‐cell response (with AST/ALT increases) ranges 4–10 weeks after its administration; it occurs with vector doses ≥ 4 × 10^11^ vector dose vg/kg (vg/kg) and its severity appears to be dose‐dependent [39, 61]. Early immune‐suppression with steroids, started soon after the detection of serum ALT increase and/or reduction of the plasma clotting factor, was safe even in persons with active HCV infection, [39, 53, 61] blunting the capsid‐specific cytotoxic T‐lymphocytes response and preserving transduced hepatocytes (and the efficacy of the therapy). In the valoctocogene roxaparvovec trial, an increase in ALT levels was detected in 115/134 participants (85.8%). Of them, 106 (79.1%) received glucocorticoids and the median treatment duration was 230 days (range, 22 to 551). Glucocorticoid‐related adverse events were reported by 71.8% of participants. At 2‐year data cut, no new safety signals emerged and no treatment ‐or drug‐related serious adverse events occurred. The results of liver biopsies taken up to 4 years after gene therapy confirmed successful transduction and showed no evidence of drug‐induced liver injury. On the other hand, data on the role of patients’, manufacturing and treatment‐related parameters are emerging from the understanding of the mechanisms of the loss of transgene expression [62, 63]. These data imply that an assessment of liver fibrosis, using diagnostic imaging techniques is required within 6 months of infusion and measurement of liver laboratory values (AST, ALT, GGT, albumin, total bilirubin and spontaneous INR) within three months [58] should be recommended. The SmPC of valoctocogene roxaparvovec [28] recommends two measurements and baseline value determination (through averaging). However, this Working Group suggests the following patient stratification after the first measurement for diagnostic appropriateness:

- Patients with out‐of‐range laboratory values and advanced liver fibrosis/cirrhosis should be classified as ineligible for GT and repeat marker testing is unnecessary.
- Patients with out‐of‐range laboratory values but without advanced liver fibrosis/cirrhosis or patients with liver markers within the normal range but suspected of advanced liver fibrosis/cirrhosis require revaluation with a second measurement of liver markers.
- Patients with values within the normal range and without advanced liver fibrosis/cirrhosis should be considered eligible for GT and repeat marker testing is not required.

Furthermore, considering the increasing prevalence of a high body mass index (BMI ≥ 30), insulin resistance, metabolic syndrome and/or diabetes mellitus and the rising trend of alcohol abuse among young populations, a steatotic liver disease (SLD) is emerging worldwide as a common cause of chronic liver disease, even in patients with haemophilia [53, 57]. Therefore, patients with SLD require a careful evaluation of the likely progression to advanced liver fibrosis/cirrhosis through liver stiffness measurement and non‐invasive tests designed to stratify the stage of the disease, predict long‐term consequences and monitor the response to diet medications [53].

At the end of the pre‐screening evaluations, patients who are ineligible for GT treatment may be referred to local psychological counselling and continue with one of the available therapies. For patients who meet the eligibility criteria, the SDM strategy is intensified to enhance the engagement of both patients and clinicians in medical decisions [53, 64, 65]. To optimize the management of patients with HA undergoing GT, it is critical to enhance patient awareness, ensure that the haemophilia specialist serves as a guide and support for the patient and that actively involves the patient and their family/caregivers in a two‐way discussion that considers the patient's expectations and concerns and tailor and personalize communication to facilitate a targeted therapeutic approach, where the patient's commitment ensures optimal pathway management. Benefits of this approach include reduced information asymmetry, informed choices before, during and after treatment, personalized treatment pathways, improved quality of life and cost optimization. SDM provides comprehensive information across multiple visits. This allows the clinicians to examine potential additional information (i.e., medical records, thorough family history) and the patient to read, digest and discuss the issue with family and friends [53]. Two or more meetings over approximately 4–6 months are recommended to share information about GT and assess the patient's interest in proceeding with the treatment. The first meeting should address the patient's unmet needs, treatment expectations and motivation. Clinicians should explain what GT is, with pros, cons and limitations and how GT can meet patient's needs. Subsequently, one or more in‐depth meetings should be scheduled, depending on the patient's needs, covering various aspects, including GT eligibility criteria, risks and benefits of short‐ and long‐term treatment, willingness to participate in long‐term data collection registries, impact on quality of life, lifestyle and post‐treatment recommendations (e.g., how long to use double barrier contraception, how long to abstain from alcohol consumption, which medications and supplements cannot be used), duration and individual variability in transgene expression and potential corticosteroid intake [28, 29, 30, 31, 32, 33, 34, 35, 36, 37, 38, 39, 40, 41, 42, 43, 44, 45, 46, 47, 48, 49, 50, 51, 52, 53, 54, 55, 56, 57, 58, 59, 60, 61, 62, 63, 64, 65, 66].

During the meetings, clinicians or other members of the multidisciplinary team (i.e., psychologists, nurses, pharmacists, physiotherapists) [53] supported by the patient advocate/patient association must adopt clear communication, which includes clear language, transmit simple and unambiguous messages and verify that the patient has understood all the information provided. Data on the development of cancer in subjects receiving GT should also be processed. Details should be provided on two patients who had been infused with valoctocogene roxaparvovec 3 and 5 years before in phase1/2 and in phase 3 studies respectively, who developed a salivary gland carcinoma and a B‐cell acute lymphoblastic leukaemia. In both cases, whole genome sequencing analysis led the trials Data Monitoring Committee to argue against such malignancies as related to the gene therapy. Same conclusions were also accomplished for other two cancers (a liver adenocarcinoma and a tonsil cancer) reported in participants of any haemophilia gene therapy trial. It will also be clarified that, based on Manufacturer's recommendations, malignancies that will eventually develop in those undergoing GT will be assessed and analysed according to a standard protocol agreed with International and Local Health Authorities [53]. Once the patient expresses the will to proceed, the Spoke Centre draws up the clinical report and refers the patient to the Hub Centre.

### 2.4 GT Prescription

In this phase, the Hub Centre confirms the patient's eligibility, obtains informed consent for the acceptance of the infusion and, in collaboration with the Spoke Centre, schedules the infusion timeline and post‐infusion monitoring activities (Figure 2). Then, the haematology specialist prescribes GT according to Regional Resolutions and obtains authorization for drug supply by filling out the AIFA Monitoring Register [67]. Subsequently, the hospital pharmacy of the Hub Centre accesses the AIFA platform to obtain drug purchase authorization and place the order. The Hospital Pharmacy at the Hub Centre takes care of drug preparation according to the SmPC of valoctocogene roxaparvovec [28] and the Good Manufacturing Practice for Pharmacy Compounded Sterile Preparations. This internal pharmacy is adequately equipped for receipt and temperature‐controlled storage of drugs and laboratories for the preparation of sterile galenic formulations according to NBP FU XII ed.

**FIGURE 2:**
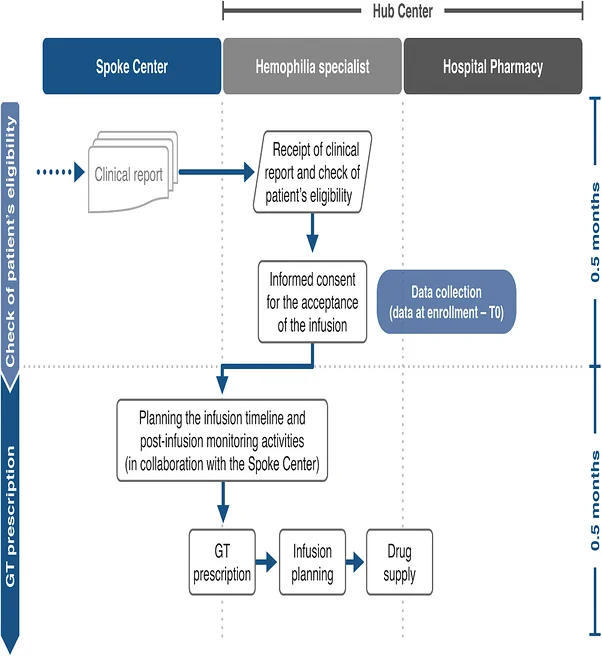
Patient journey: Gene therapy prescription. GT, Gene Therapy.

At this point of the journey, patient data should be collected and recorded in a national disease registry and eventually transferred to the WFH GTR [68, 69]. Currently, data collection occurs through the AIFA Monitoring Register [67], which verifies the appropriateness of prescriptions in the pre‐infusion phase. However, it is essential to integrate these data with safety, efficacy and duration of action data in the post‐administration period over a short‐ to long‐term.

### 2.5 GT Administration and Post‐Infusion Follow‐up

GT administration and post‐infusion follow‐up are managed by the Hub Centre in collaboration with the Spoke Centre (Figure 3). The Hospital Pharmacy at the Hub Centre handles receipt, stores, handles and governs advanced therapy medicinal products (ATMPs) and oversees the Good Manufacturing Practice for: 1) Pharmacy Compounded Sterile Preparations and 2) drug preparation (in this specific setting, according to the SmPC of valoctocogene roxaparvovec) [28]. For innovation drugs, insurance, financial considerations and regulatory rules for pharmacies of the hub Centres in Italy are based on regional fundings and regimens. This was the funding model for the 8 Italian regions where valoctocogene roxaparvovec was employed. The SDM process should be repeated at this phase to support the patient during the infusion and subsequent monitoring.

**FIGURE 3:**
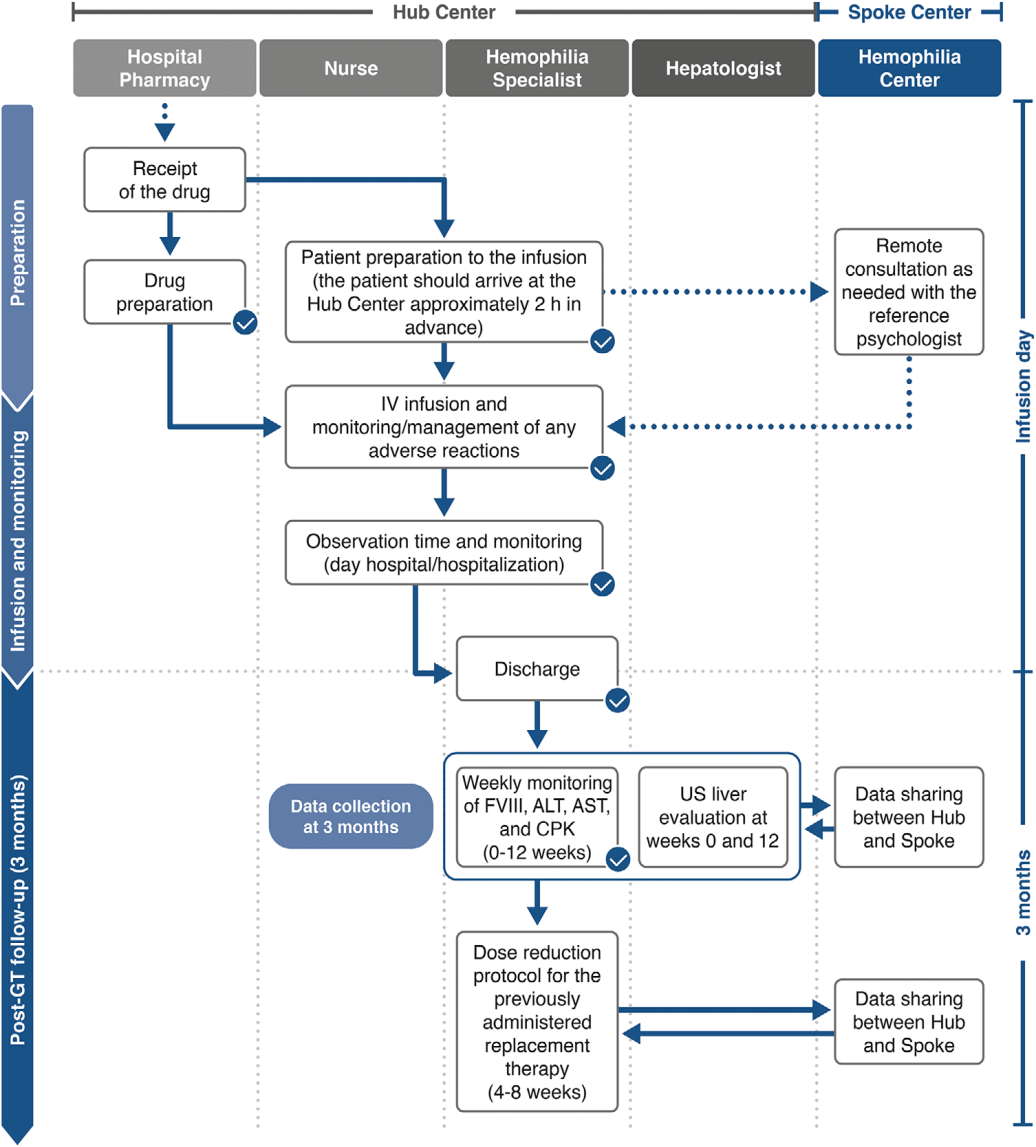
Patient journey: Gene therapy administration and post‐infusion follow‐up (<3 months). *Note*: The boxes with the check marks contain indications from the summary of product characteristics. ALT, Alanine Aminotransferase; AST, Aspartate Aminotransferase; CPK, Creatine phosphokinase; FVIII, Factor VIII; GT, gene therapy; US, ultrasound.

On the day of GT infusion, the patient should arrive at the Hub Centre approximately 2 h in advance. During this period the patient can access remote consultation with the reference psychologist. The infusion duration can range from 2 to 5 h (or longer for patients > 100 kg), depending on the volume and rate of infusion and the patient's response [28]. In the event of adverse reactions occurring during the infusion, it is recommended to slow or suspend the infusion, administer pharmacological intervention and prolong observation. Special precautions should be taken before handling or administering the medicinal product: According to the Manufacturer's instructions, (1) the medicinal product contains genetically modified organisms (GMOs) and each vial is for single use only; (2) during the preparation, administration and disposal of the valoctocogene roxaparvovec solution and of materials that have come into contact with the solution (solid and liquid waste), personal protective equipment (including a lab coat, safety goggles, a mask and gloves) must be worn; (3) valoctocogene roxaparvovec must not be exposed to ultraviolet (UV) disinfection lamp light and (4) should be prepared using aseptic technique. Finally, when assembling the infusion set, the treated must know whether the surfaces of the components in contact with the valoctocogene roxaparvovec solution are made of compatible materials (e.g., infusion pump syringes: polypropylene (body), infusion set, polyethylene tip; Infusion catheter, polyurethane‐based polymer; needles: stainless steel).

According to the SmPC [28], infusion‐related reactions typically occur within a median onset time of 1 h from the start of the infusion, have a median duration of 1 h and can manifest in various ways: cutaneous, mucosal, respiratory, gastrointestinal, cardiovascular, musculoskeletal, pyrexia, cold shivers and chills. Summary of Product Characteristics also states that treatment of infusion‐related reactions involves systemic antihistamines, corticosteroids, antiemetics and epinephrine. An anaesthesiologist should be pre‐alerted to manage any serious adverse reactions. Infusion can be managed in a day‐hospital setting with subsequent observation time evaluated on a case‐by‐case basis; the patients should be observed for 1–2 h after the infusion and if no side effects occur, they could be discharged.

Upon discharge, clinicians should inform the patient about new or recurring reaction management and remind the patient of the behaviours to adopt regarding alcohol consumption, double barrier contraception, concurrent medication and supplements, physical activity and adherence to follow‐up appointments. It is essential to inform the patient that the monitoring schedule is tight and that it must be respected.

During the first 12 weeks post‐infusion, the patient must be monitored to promptly address the onset of adverse events. During this period, the patient remains in the care of the Hub Centre, which periodically shares the patient's information and data with the Spoke Centre.

During the first 12 weeks post‐infusion and subsequently until week 26, weekly monitoring of FVIII levels and liver markers (ALT, AST) and CPK is recommended. FVIII activity should be measured using chromogenic substrate assays. Liver ultrasound is recommended at 0‐ and 24‐week post‐infusion; and subsequently every 6 months. However, as clarified throughout the manuscript and in Figure 1, additional scans (e.g., at 12 weeks) should be considered for example, if ALT and AST persist elevated also for other reasons. Between the fourth and eighth post‐infusion week, dose reduction protocol for the previously administered replacement therapy.

Following administration of valoctocogene roxaparvovec, the patient could experience an increase of liver enzymes and, in some cases, it has been associated with decreased expression of the FVIII transgene protein. ALT and FVIII activity levels should be monitored after infusion and corticosteroid treatment should be instituted in response to ALT elevations as needed, to control hepatic reactions and prevent or mitigate a potential reduction in transgene expression [25]. Consistent with the GENEr8‐1 study [26], an increase in ALT of at least 1.5‐fold from baseline or to above ULN is the threshold at which immunosuppression should currently be initiated [39, 61]. According to this journey, based on clinical experience, ALT measurements are repeated twice and, when consistent enhancements are documented with loss of VIII expression, patients with valoctocogene roxaparvovec‐associated ALT elevation should receive prednisone 60 mg/day (or equivalent dose of another corticosteroid) for 2 weeks. If ALT continues to rise or has not improved after 2 weeks, the corticosteroid dose should be increased to a maximum of 1.2 mg/kg. If ALT levels remain stable after 2 weeks and/or earlier when ALT levels start to decline, the daily dose can be gradually tapered [28]. However, caution should be used because methylprednisolone administration may be associated with drug‐induced liver autoimmune‐like hepatitis. Prolonged administration of high corticosteroid doses is not considered appropriate, so in addition to further workup for alternative causes of ALT elevations, other immunosuppressants may be considered if high‐dose prednisone proves to be ineffective [59, 60, 61]. These steps need a close collaboration with expert hepatologists of the HUB centre.

At this point of the journey, patient data (data at 3 months – T1) should be collected and recorded in a regional and national disease registry and then eventually transferred to the international data centre [5]. Regardless of details that still need to be elucidated with respect to the handling of the Italian national GT Registry of Haemophilia, in the first year after therapy, ALT levels are monitored once/week to detect elevations ≥ 1.5 × ULN, which may require immunosuppressant therapy together with abdominal ultrasound and liver stiffness measurements by transient elastography.

At the end of the first 12 weeks post‐infusion, the patient returns to the care of the Spoke Centre (Figure 4). At this point in the journey, patient data should be collected and recorded in a national disease registry.

**FIGURE 4:**
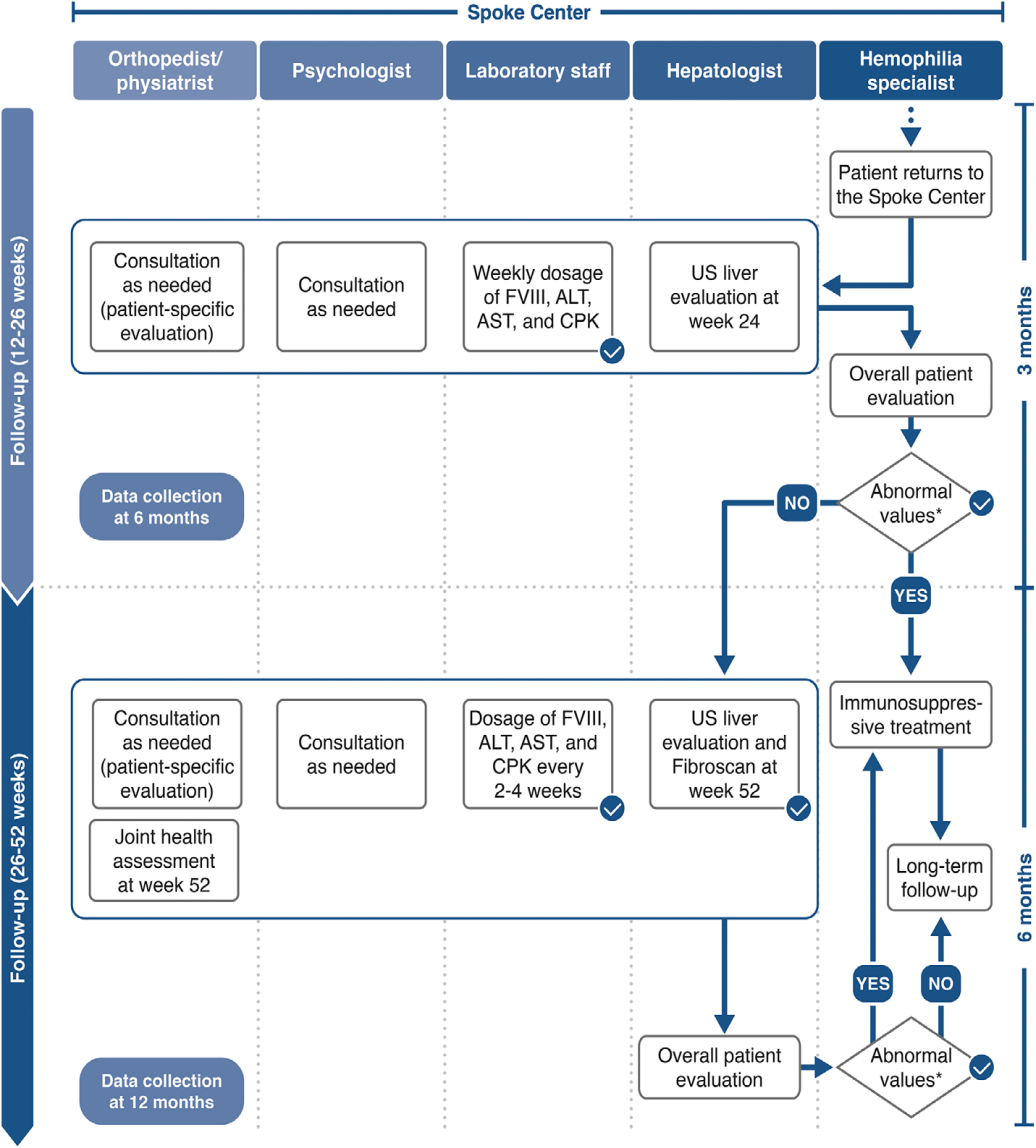
Patient journey: Short‐term post‐gene therapy follow‐up (≤1 year). *ALT > 1.5 Baseline Value; ALT > ULN; FVIII Activity Reduction. *Note*: The boxes with the check marks contain indications from the summary of product characteristics. ALT, Alanine Aminotransferase; AST, Aspartate Aminotransferase; CPK, Creatine phosphokinase; FVIII, Factor VIII; ULN, upper limits of normal; US, ultrasound.

As already reported, it is recommended weekly dosage of FVIII activity level and hepatic and muscular markers (ALT, AST and CPK) until week 26. Like in the first liver‐directed GT study in HB with the rAAV2 vector [59], studies with rAAV8 (a vector with a stronger hepatic tropism than rAAV2) and with rAAV5 (the serotype with the lowest prevalence of NAbs in the general population) [70, 71] document dose‐dependent cytotoxic T‐cell response against transduced hepatocytes in some infected persons [72]. This led to lysis of transduced cells (together with an asymptomatic serum alanine aminotransferase [ALT] increase called ‘transaminitis’) and loss of transgene expression [73]. These measurements are crucial for the early detection of any elevation in transaminase levels, understanding their origin (whether hepatic or muscular) and intervening accordingly. Variations or elevations in transaminase levels can be related to lifestyle changes (e.g., dietary variations, alcohol or medication intake, etc.) or underlying conditions. Therefore, the haemophilia specialist, in collaboration with the hepatologist, evaluates the patient's overall condition, performs a differential diagnosis, including acute infection with major and minor hepatotropic viruses and assess the clinical severity in terms of impact on liver function. If at week 24, ALT levels are increased to 1.5 times the baseline value, ALT exceeds the upper limit of normal, or FVIII activity reduction is present, treatment with immunosuppressive should be considered [38], better along with proton pump inhibitors to mitigate the risk of gastritis and gastric bleeding. If steroid therapy is prolonged, osteoporosis prophylaxis with calcium and vitamin D3 should be considered, with one exception [47] there is no specific guideline available yet.

As already reported, corticosteroid treatment should be instituted to control hepatic reactions and prevent or mitigate a potential reduction in transgene expression [28].

During this period, a psychological consultation may be requested if there are specific elements suggesting potential benefit for the patient, while an orthopaedic evaluation is recommended annually and whenever the patient reports signs or symptoms warranting further assessment.

If none of these events occur, FVIII and hepatic marker assessments are recommended every 2–4 weeks for the next 26 weeks [28], with the possibility, in specific cases, to plan a closer monitoring frequency (every 1–2 weeks) with the hepatologist.

ALT fluctuations are common and can be caused by viral infections, food or drug toxicity, intense muscle activity and other conditions. These should be distinguished from ALT elevations due to AAV vector gene therapy‐related toxicity which may require corticosteroids treatment.

At 12 months post‐infusion, annual assessment of joint status, liver evaluation through ultrasound and FibroScan and administration of the quality‐of‐life questionnaire are recommended. It is suggested to use an ad hoc questionnaire, designed in a semi‐structured format yet framed within predefined grids to ensure the collection of measurable results [53]. If during the 25 weeks period [13, 24, 25, 26, 27, 28, 31, 32, 33, 34, 35, 36, 37, 38, 39, 40, 41, 42, 43, 44, 45, 46, 47, 48, 49, 50, 51, 74], ALT levels increase to 1.5 times the baseline value or ALT levels exceed the upper limit of normal or FVIII activity reduction is present, immunosuppressive treatment is required to control hepatic reactions and prevent or mitigate a potential reduction in transgene expression [66].

During the 12 months or until sperm is clear from vector shedding (as defined according to literature data), double barrier contraception needs to be used.

## 3 Long‐Term Post‐GT Follow‐up (>1 Year—Up to 15 Years)

The long‐term follow‐up process is managed by the Spoke Centre (Figure 5). Patient data should be collected and recorded in a national disease registry at 18 (T4) and 24 (T5) months and then annually for 15 years after gene transfer.

**FIGURE 5:**
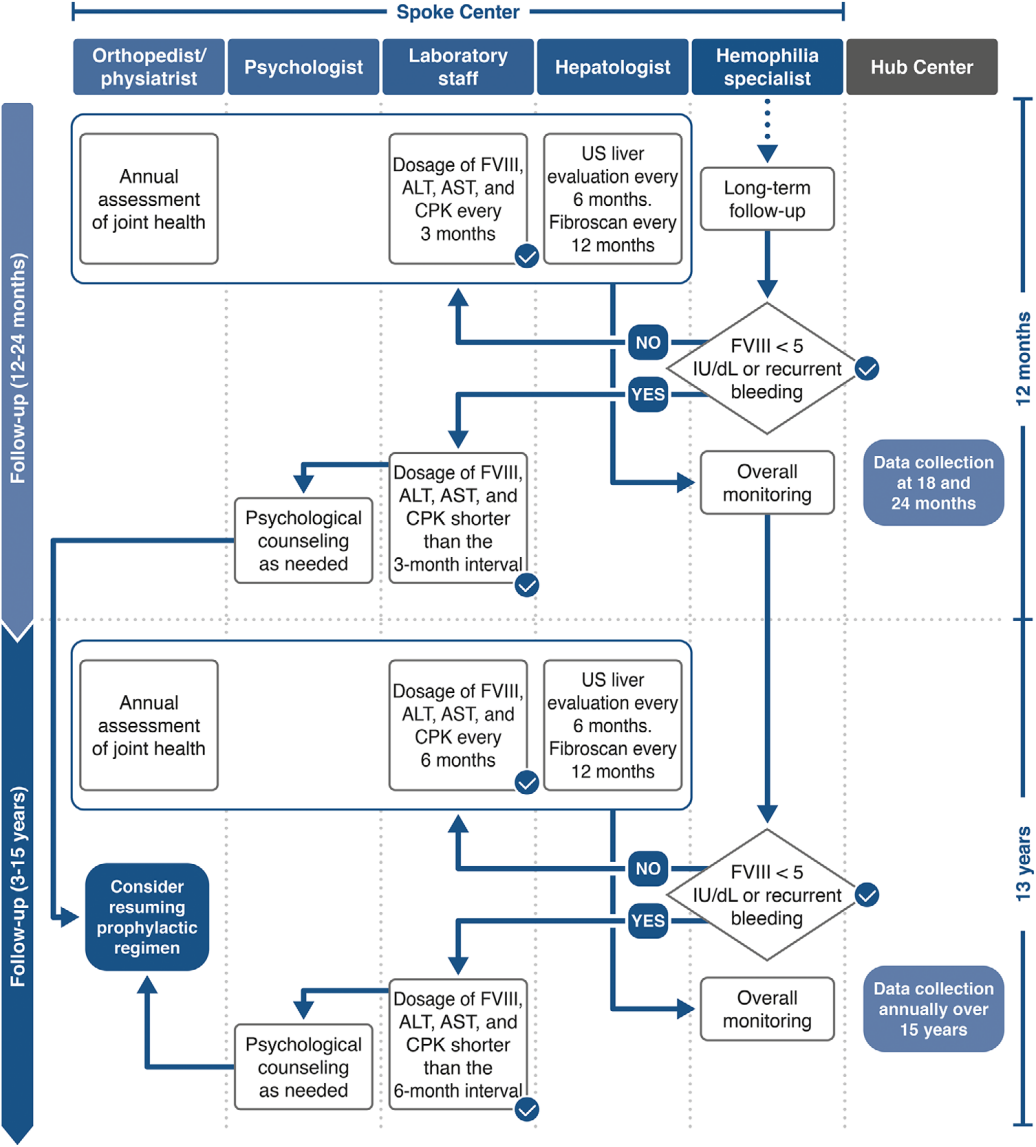
Patient journey: Long‐term post‐gene therapy follow‐up (>1 year—up to 15 years). The boxes with the check marks contain indications from the summary of product characteristics. ALT, Alanine Aminotransferase; AST, Aspartate Aminotransferase; CPK, Creatine Phosphokinase; FVIII, Factor VIII; US, Ultrasound.

If at the beginning of the long‐term follow‐up (12 months), FVIII reduction is not below 5 IU/dL and there are no signs of bleeding, measurement of FVIII and liver markers is recommended every 3 months. Joint health assessment should be performed every 12 months; liver monitoring should continue through semi‐annual ultrasound to screen for hepatocellular carcinoma and an annual FibroScan examination to monitor for fibrosis [57].

If at the beginning of the long‐term follow‐up period (12 months), FVIII reduction is below 5 IU/dL or there is evidence of recurrent bleeding, the measurements of FVIII and liver markers should be shorter than the 3‐month interval. As recommended by the WFH guidelines, patients with a severe phenotype (including patients with moderate haemophilia with a severe phenotype) must be sufficiently prophylaxed to prevent bleeds [5]. Therefore, with the support of a psychologist, for such patients it may be appropriate to resume an individualized prophylactic regimen considering patient bleeding phenotype, joint status, individual pharmacokinetics and patient self‐assessment and preference [5]. It is important to underline that lab measurement using both one‐stage assay and chromogenic assay needs to be associated with information on clinical conditions of the patient, if the patient is at risk of bleeding prophylaxis should be stated again.

If at year 3, FVIII reduction is not below 5 IU/dL and there are no signs of bleeding, FVIII and liver marker measurements are recommended every 6 months. A joint health assessment should be scheduled every 12 months; liver monitoring should continue through semi‐annual ultrasound and annual FibroScan examination. If an FVIII reduction is observed below 5 IU/dL or there is evidence of recurrent bleeding, the measurements of FVIII and liver markers should be shorter than the 6‐month interval. As reported above, it may be appropriate, with the support of a psychologist, to resume a prophylactic regimen [5].

## 4 Discussion

GT introduces a revolutionary approach to managing patients with HA, offering the potential for a functional cure by facilitating the continuous expression of FVIII. While this innovative approach marks significant progress, several challenges must be addressed before integrating GT into clinical practice.

The present document serves as a blueprint for supporting clinicians in the management of patients with HA treated with GT in Italy. It attempts to provide a general pathway to be implemented at a regional level and to be adapted to each centre.

A SDM approach, multidisciplinary team and data collection are the main requirements for the new pathway.

First, there is a need to enhance awareness, communication and education regarding the therapeutic potential and modalities of GT. Objective, transparent and regularly updated information should be disseminated appropriately and understandably, with the support of patient advocate/patient organizations. The goal is to empower them to actively participate in their care. In this context, the SDM approach is effective in increasing the involvement of both patients and clinicians in medical decisions while improving the quality of care [53].

In accordance with the UK gene therapy guidance and the WFH gene therapy recommendations [29, 30], effective care for haemophilia necessitates management by a multidisciplinary team, including haemophilia specialists, hepatologists, orthopaedics, psychologists/counsellors, physiatrists/physiotherapists, nurses, hospital pharmacists and laboratory staff. All team members must be adequately trained to ensure patients receive consistent, up‐to‐date information on GT.

The identification and formalization of a management pathway for patients with severe HA, undergoing GT, is one of the pillars for achieving economic and financial sustainability. GT in HA is an innovative treatment; therefore, the management model must be understood as a flexible framework intended to evolve based on data and experience accumulated over time.

Key actions include training the multidisciplinary team on GT, exploring all issues related to patient management according to specific roles and phases of the pathway, sharing experiences among centres and multidisciplinary members and ensuring an effective SDM approach through a unified and well‐defined communication strategy. Moreover, it is essential to offer training courses specifically designed for hospital pharmacists, enabling them to delve into the intricacies of pharmacological (such as pharmacokinetics, pharmacodynamics and interactions) and practical aspects (including supply, storage and preparation) related to GT.

Current data collection through the AIFA Monitoring Register [67] should be implemented with safety, efficacy and duration of action data in the post‐administration period data over a short‐ to long‐term.

### 4.1 Limitations

The present journey has been successfully employed in 22 AAV‐based GT in Italy and no evidence‐bases limitation has emerged. As clearly stated, it needs to be amended for other GT products and non‐Italy settings. The Manufacturer sponsorship and the product‐specific scope should also be considered.

## 5 Conclusion

The present document suggests a new operational and organizational model that must consider the peculiarities of the journey for patients with HA treated with GT in Italy. A patient‐centred approach is required, focusing on patients’ needs and expectations

To enhance the HA patient journey in GT, actions include increasing patient awareness through SDM approach, defining team roles through a shared protocol and promoting structured collaboration between Hub and Spoke centres via regular meetings for in‐depth case analysis during GT.

The selection of reference centres according to a Hub and Spoke network and the clarification of activities, personnel and resources involved can help achieve important economies of scale [68].

The establishment of multidisciplinary teams, properly trained, within both the Hub and Spoke centres and continuous communication and collaboration are essential to optimize patient management, accelerate diagnostic and therapeutic choices and ensure timely intervention.

The establishment of a national registry is essential, with the subsequent merging of data into the international GT Registry (GTR) developed by the World Federation of Hemophilia (WFH) [64].

Although gene therapy with Valoctocogene roxaparvovec is no longer commercially pursued worldwide, nearly 50 patients with severe HA have been treated in USA, Germany and Italy. The clinical and operational strategies developed in each country could provide meaningful knowledge on dosing approaches, safety management, regulatory pathways and manufacturing logistics and this could possibly carry over to newer therapies.

## Author Contributions

Definition of the patient journey and critical revisions of the manuscript were carried out by all authors. Giovanni Di Minno and Flora Peyvandi coordinated and revised the accuracy manuscript. Final approval for the version to be published was obtained from all authors. All authors have read and approved the final manuscript.

## Funding

This study was supported by BioMarin Pharmaceutical.

## Ethics Statement

This manuscript presents a roadmap and organisational plan and does not involve human participants, personal data, or animal subjects. As such, ethical approval and informed consent were not required. The person in charge of the roadmaps is the chairman of the Health Direction, here present among authors (AB).

## Conflicts of Interest

Giovanni Di Minno is a member of speaker bureaus for BioMarin, Bayer, CSL Behring; and is an ad‐hoc speaker/consultant for BioMarin. Alessandra Borchiellini served on an advisory board for Takeda, Novo Nordisk, Grifols, Viatris, Roche, Amgen, Sobi, Pfizer, Sanofi; has received payment or honoraria for lectures, presentations, speaker's bureaus from Takeda, Novo Nordisk, Grifols, Viatris, Roche, Amgen, Sobi, Pfizer, Sanofi. Anna Borrelli declares no competing financial interest. Andrea Buzzi declares no competing financial interest. Giancarlo Castaman served on an advisory board for Bayer, BioMarin, Bioviiix, CSL Behring, Kedrion, LFB, Novo Nordisk, Pfizer, Roche, Sobi and Takeda; has received payment or honoraria for lectures and speaker's bureaus from Bayer, BioMarin, Bioviiix, CSL Behring, Kedrion, LFB, Novo Nordisk, Pfizer, Roche, Sobi and Takeda. Michele Cecchi declares no competing financial interest. Raimondo De Cristofaro has received research funding from Sanofi; has received payment or honoraria for speaker's bureaus from Pfizer, Roche, BioMarin, Bayer, Sobi and Kedrion; has acted as paid consultant for Roche Diagnostic. Isabella Garagiola declares no competing financial interest. Vincenzo La Mura has acted as paid consultant for BioMarin, CSL Behring and Pfizer; has received payment for speaker's bureaus from BioMarin, Gore and Alfa‐sigma; received support travel grant from Bayer, Takeda and Sanofi. Angelo Lupi declares no competing financial interest. Giuseppe Malcangi has acted as paid consultant to Bayer, Takeda, Novo Nordisk, BioMarin, Pfizer, CSL Behring and Sobi. Maria Elisa Mancuso has acted as paid consultant and receive payment for speaking and advisor from Bayer, BioMarin, CSL Behring, Kedrion, LFB, Novo Nordisk, Octapharma, Pfizer, Regeneron, Roche, Sanofi, Sobi and Takeda. Maria Francesca Mansueto has worked as a consultant and speaker for Novo Nordisk, Sobi, Kedrion, Roche and BioMarin. Cristina Santoro served on an advisory board and received payment for speaker's bureaus BioMarin. Rita Carlotta Santoro served on an advisory board, has received payment or honoraria for lectures, presentations, speaker's bureaus, manuscript writing, or educational event and received support for attending meetings and/or travel from Bayer, BioMarin, Roche and SOBI. Luigi Piero Solimeno has received speaker fee from Sobi. Gaia Spadarella declares no competing financial interest. Annarita Tagliaferri served on an advisory board for Bayer; has received speaker fee from Novo Nordisk and Sobi; served on an editorial project for BioMarin. Ezio Zanon has acted as paid consultant to Bayer, Kedrion, CSL Behring, Novo Nordisk, Sobi, Pfizer, BioMarin and Takeda. Flora Peyvandi has received honoraria for participating in advisory board organized by CSL Behring, BioMarin, Roche, Sanofi, Sobi, Pfizer and educational meetings/symposia organized by Takeda, Sanofi and Sobi.

## Data Availability

No new or individualised data were generated or analysed in this study. Therefore, a data availability statement is not applicable.
